# Association between cardiovascular disease and non-melanoma skin cancer: The mediation effect of obesity and inflammation

**DOI:** 10.1371/journal.pone.0343992

**Published:** 2026-03-23

**Authors:** Xin Zhang, Zhe Gao, Ying Miao, Xin-Gang Wu

**Affiliations:** 1 Department of Dermatology, Hangzhou Third People’s Hospital, Hangzhou, China; 2 Department of Dermatology, The Third Affiliated Hospital of Soochow University, Changzhou, China; Hamadan University of Medical Sciences, School of Public Health, IRAN, ISLAMIC REPUBLIC OF

## Abstract

**Background:**

Although the association between CVD and various cancers has been extensively studied, its relationship with NMSC remains ambiguous. Previous studies have shown that cardiovascular disease (CVD) is an independent risk factor for tumorigenesis. However, the relationship between CVD and non-melanoma skin cancer (NMSC) is unclear. The aim of this study is to investigate the potential relationship between CVD and NMSC and whether obesity and inflammation mediate the association.

**Methods:**

7424 participants from the National Health and Nutrition Examination Survey (NHANES) from 2015 to 2018 were included. Diagnosis of CVD and NMSC was determined by questionnaire combined with self-reported. Inflammatory markers and obesity indices assessed were SIRI, SII, BMI, and WWI. Logistic regression and Pearson correlation analyses were applied to investigate the relationship between the above key variables.

**Results:**

Logistic regression results showed that CVD was a risk factor for NMSC (OR: 1.83, 95% CI: 1.01 ~ 3.34, p = 0.048); however, there was no statistically significant association between CVD subgroups and NMSC. In addition, SIRI, BMI, and WWI partially mediated the association between CVD and NMSC (p < .001), but SII did not alter the relationship (p > 0.05). Bootstrap test confirmed the stability of the results of the mediation analysis.

**Conclusion:**

CVD increases the risk of developing NMSC, and obesity and inflammation partially mediate the relationship. Weight loss and control of inflammation may be beneficial in reducing the prevalence of CVD and NMSC.

## Introduction

Non-melanoma skin cancers (NMSCs) are the most common skin cancers. 99% of NMSCs are basal cell carcinomas (BCCs) and squamous cell carcinomas (SCCs) [1]. Other rare tumours, such as sebaceous gland carcinoma and apocrine sweat gland adenocarcinoma, are also included [2]. The etiology of NMSC is likely to be related to exposure to UV radiation and increased zone depletion [3], but the exact pathogenesis is unclear. In recent years, the incidence of NMSC has been gradually increasing due to population aging, and the growing disease burden constitutes a serious global healthcare problem [4,5]. In order to reduce the disease burden, understanding the risk factors of cancer will enable us to avoid the development of NMSC effectively.

Cardiovascular disease (CVD) is a group of diseases affecting the heart and blood vessels [6], including stroke, angina, congestive heart failure (CHF), coronary heart disease (CHD), and heart attack [7]. Both cancer and CVD are recognized as serious public health issues due to their high global prevalence and mortality rates. Despite their apparent disassociation, a US study indicated that the co-prevalence of cancer and CVD was as high as 16.2% [8]. A growing number of studies suggested that CVD may be a risk factor for cancer development [9–12]. In a previous mouse model, researchers found that mice with failing hearts promoted tumor cell proliferation through the protein SerpinA3 secreted by cardiomyocytes [10]. In another prospective cohort study, CVD markers including GDF15, SDF1, GRN and FGF23 were found to be most substantially correlated with cancer incidence [13]. Meanwhile, cancer survivors were linked to the risk of developing CVD, thus suggesting a new area of research: cardio-oncology [13]. In a large community-based prospective study, researchers found that adult cancer survivors were at significantly higher risk of developing CVD (especially CHF) compared to individuals without cancer [14]. A matched cohort study based on the UK Biobank also discovered that patients newly diagnosed with cancer were at increased risk of developing multiple types of CVD [15]. The aforementioned studies provide evidence for a bidirectional relationship between CVD and cancer. However no one has yet studied the relationship between CVD and NMSC, and it is reasonable to assume that there is also a correlation between CVD and NMSC. The exact mechanism of action between CVD and cancer (including NMSC) is currently unclear, and there may be shared risk factors and pathogenesis for both. Based on previous studies, this association may be explained by inflammation and obesity [13,16,17]. Obesity is a risk factor for many chronic diseases, and inflammation is a pathophysiologic pathway shared by many chronic diseases.

It is well known that obesity is one of the most important risk factors for CVD [16,18], which triggers systemic chronic inflammatory responses and increases oxidative stress to promote the development of CVD [19]. Furthermore, a Canadian study observed a correlation between obesity and the prevalence of NMSC, with the link being stronger in regions with less UV exposure [20]. The above evidence suggests that inflammation and obesity play an important role in the pathogenesis of CVD as well as NMSC and they may also have a significant impact on the relationship between CVD and NMSC. Gaining insight into this mediation role may help us better understand the pathophysiology of both diseases and provide more effective strategies for the prevention of CVD and NMSC.

In recent years, some novel indicators of obesity and inflammation have attracted attention. Body mass index (BMI) is a commonly used index to assess obesity, but it is unable to distinguish between muscle mass and fat mass [21]. Therefore, we introduced the weight-adjusted waist circumference index (WWI), which standardizes the WC with body weight, incorporating the advantages of the WC while weakening the relationship with BMI [22]. The WWI not only distinguishes between components of muscle and fat mass but is also a measure of central obesity [23]. The systemic immune inflammation index [24] and systemic immune response to inflammation index (SIRI) are novel metrics derived from complete blood count, which have been widely used in studies assessing the association between chronic inflammatory states and a variety of human diseases, including cancers, metabolic disorders, and inflammation [25,26].

Therefore, this study aimed to explore the relationship between CVD and NMSC and determine whether BMI, WWI, SII, and SIRI play a role by analyzing adult data from the National Health and Nutrition Examination Survey (NHANES).

## Methods

### Population and data sources

**Fig 1:**
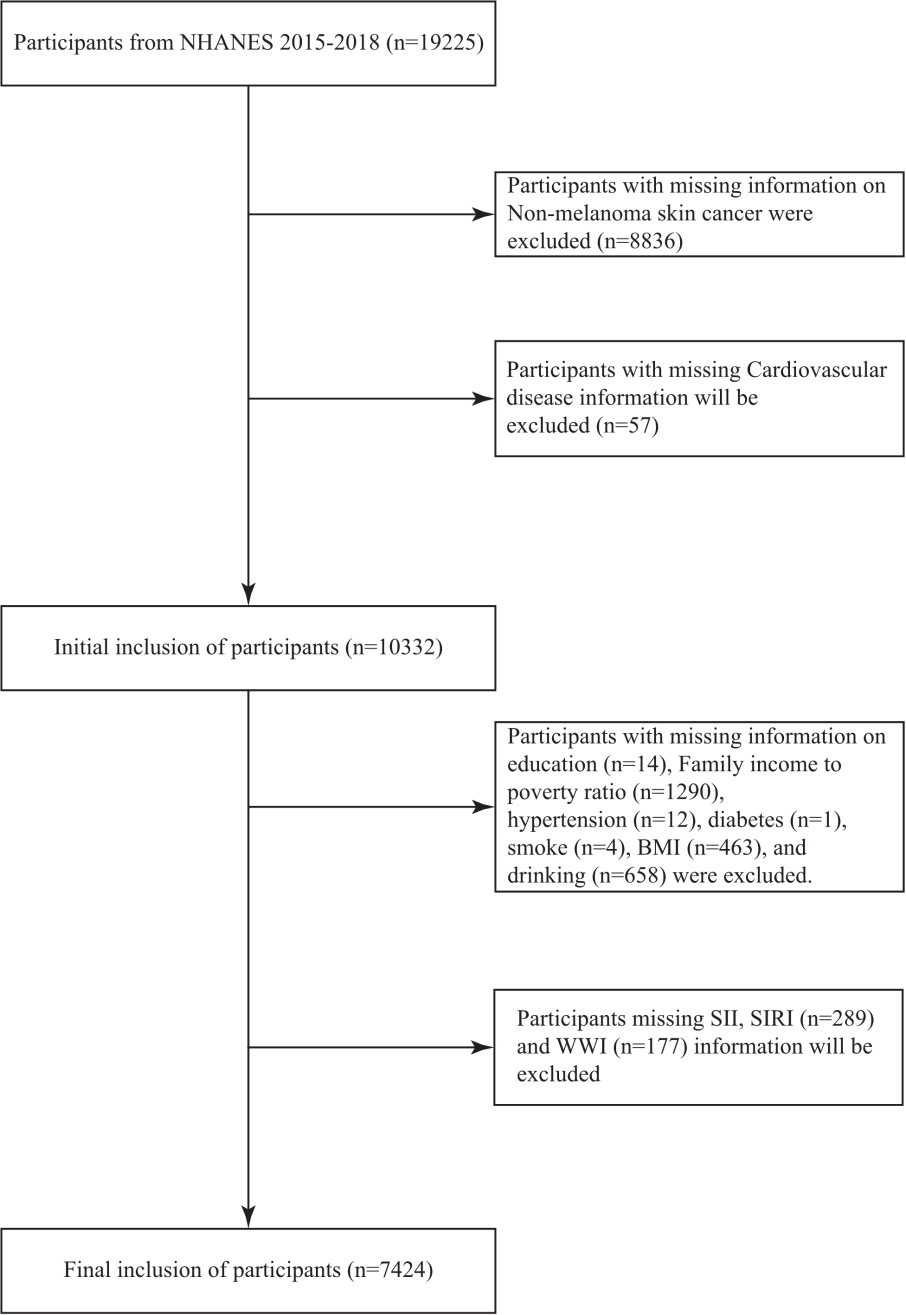
The flow chart shows the study design and exclusion criteria.

Data from the 2015−2018 National Health and Nutrition Examination Survey (NHANES) were used in this study, selected for complete availability of inflammatory markers and to precede COVID-19 influences on health trends. NHANES is a continuous cross-sectional observational study that collects representative health and nutrition information from a noninstitutionalized population in the United States. All participants provided written informed consent, which was approved by the National Center for Health Statistics Institutional Review Board (Protocol #2011−17). The NHANES questionnaires for CVD and NMSC have been validated for reliability in prior studies, showing high agreement with medical records (kappa > 0.7) [27,28]. The study population for this article consisted primarily of 19,225 U.S. adults (≥20 years of age) from 2015 through 2018. After excluding those missing important data (Fig 1), 7424 subjects were ultimately included.

### Ethics approval and consent to participate

The study was carried out in accordance with the Declaration of Helsinki. The NCHS study Ethics Review Board authorized all study protocols, and survey participants provided signed informed consent.

### Definition of non-melanoma

The prevalence of NMSC among participants was collected through the following questions in the questionnaire: “{Have you/Has the standardized patient} ever been informed by a doctor or other health professional that {you/she/he} had skin cancer?” and “How old were you when you were first informed that you had skin cancer?”. Answers were used to collect the prevalence of NMSC, which was in accordance with previous studies [27].

### Definition of CVD

Consistent with a previous study [28], the diagnosis of CVD was determined based on the Medical Conditions Questionnaire combined with the participant’s self-reported. They were asked the following question: “Have you ever been told by a doctor or other health professional that you have CHF/CHD/angina/MI/stroke?”. If the answer was yes, they were categorized as having CVD. CVD subgroups were classified as follows: congestive heart failure (CHF), coronary heart disease (CHD), angina, myocardial infarction (heart attack), and stroke, based on self-reported diagnoses to specific questions for each condition.

### Definitions of SIRI, SII, BMI and WWI

BMI and WWI are indicators of obesity. BMI is calculated as weight (kg) divided by the square of height (m), and WWI is a new indicator of central obesity that standardizes waist circumference (WC) and body weight to distinguish between fat and muscle mass. This index is calculated as WC (cm) divided by the square root of weight (kg) [22]. To ensure data accuracy, weight and WC were measured by medical staff using a standardized instrument. The systemic immune-inflammation index and systemic inflammation response index (SIRI) is a new index based on blood counts to reflect the systemic inflammatory status. SII is calculated as platelet count (PLT) multiplied by the neutrophil-to-lymphocyte ratio (NLR). SIRI is computed as neutrophil count multiplied by the monocyte-to-lymphocyte ratio [26].


$$SII=\;\frac{PLT\times Neutrophils}{\text{Lymphocytes}}$$

$$SIRI=\;\frac{\text{Monocyte}\times Neutrophils}{\text{Lymphocytes}}$$

### Covariates

According to previous studies, potential influencing factors were determined, including Age, Sex, Race (Mexican, Other Hispanic, Other Race), Education (<9th grade, 9–11th grade, High school diploma/GED, Some College/AA degree, > College graduate), Family income to poverty ratio, Drink alcohol, Smoking status, Hypertension and Diabetes. Hypertension was defined as “Ever told you had high blood pressure” and “Now taking prescribed medicine for HBP” [29]. Diabetes was defined as “Doctor told you had diabetes”, “Two Hour Glucose(OGTT)(mmol/L)≥11.1”, and” Fasting Glucose(mmol/L)≥7.0” [30]. Smoking status was divided into current non-smokers (never smoked or quit smoking for more than 1 year) and current smokers (currently smoking, or have smoked for more than one day in the past 30 days, or wake up smoking, or have smoked more than two cigarettes per day after quitting smoking) [31]. Drinking status is categorized as never drinking (less than 12 drinks in lifetime) and current drinking (at least 12 drinks per year or more than 6 drinks in the past 12 months) [32],

### Statistical analysis

The R software package (version 4.4.1) was used for all statistical analyses. In order to more accurately reflect the overall population, we used the “survey” package to weigh the population to account for the complex sampling design of NHANES. Weighted analysis is a statistical method that assigns different weights to observations in a sample to reflect their importance and representativeness in the overall data set. NHANES weighting and methodology are described on its official website and in previous research in detail [33,34]. The initial sample for this study consisted of 7424 participants, weighted as shown in Table 1, representing a weighted population of 173,312,738 U.S. adults. Continuous variables are expressed as means and standard deviations (SD), while categorical variables are expressed as frequencies and percentages. Independent t-tests were used for continuous variables and chi-square tests were used for categorical variables. Logistic regression models were used to analyze the association between CVD and NMSC prevalence and to adjust for potential confounders. The covariate adjustment hierarchy was: Model 1 (unadjusted); Model 2 (adjusted for sex, age, race/ethnicity, education, family income to poverty ratio, BMI); Model 3 (Model 2 + drinking status, smoking status, hypertension, diabetes); Model 4 (Model 3 + SIRI, SII, WWI). Subgroup analyses were used to explore the effect of different demographic characteristics on the association between CVD and NMSC. Subsequently, the correlations between CVD, NMSC, SII, SIRI, WWI, and BMI were investigated using Pearson’s analysis, with categorical variables (CVD, NMSC) coded as binary (0/1) for point-biserial correlations. Mediation analyses were performed using the “mediation” R package to assess the mediating effects of SII, SIRI, WWI, and BMI. Finally, effect sizes were evaluated via 5000 bootstrap iterations and 95% confidence intervals were obtained [35]. Bootstrap testing used bias-corrected accelerated intervals with 5000 iterations. The mediating effect was significant when the 95% confidence interval did not include zero [36]. All statistical tests were two-sided and p < 0.05 was considered statistically significant.

**Table 1:** Characteristics of the NHANES 2015–2018 participants.

Characteristics		Overall N = 173,312,738	No Non-melanoma skin cancer N = 166,935,488	Non-melanoma skin cancer N = 6,377,251	p
Sex^b^, n(%)	7,424				0.766
FeMale		3,778 (51%)	3,697 (51%)	81 (49%)	
Male		3,646 (49%)	3,544 (49%)	102 (51%)	
Age(year)^a^, Mean ± SD	7,424	46.84± (16.54)	46.15± (16.28)	64.85± (12.65)	<0.001
Age group^b^, n(%)	7,424				<0.001
<=49		3,793 (56%)	3,780 (57%)	13 (11%)	
> 49		3,631 (44%)	3,461 (43%)	170 (89%)	
Race/ethnicity^b^, n(%)	7,424				<0.001
Mexican		1,164 (8.8%)	1,160 (9.1%)	4 (0.4%)	
Other Hispanic		816 (6.1%)	810 (6.3%)	6 (1.0%)	
Other Race		5,444 (85%)	5,271 (85%)	173 (99%)	
Education^b^, n(%)	7,424				0.012
< 9th grade		648 (3.9%)	647 (4.1%)	1 (<0.1%)	
9–11th grade		814 (7.5%)	797 (7.6%)	17 (5.5%)	
High school diploma/GED		1,743 (25%)	1,703 (25%)	40 (18%)	
Some College/AA degree		2,357 (32%)	2,287 (32%)	70 (35%)	
≥ College graduate		1,862 (32%)	1,807 (32%)	55 (42%)	
Family income to poverty ratio^a^, Mean ± SD	7,424	3.06± (1.64)	3.04± (1.64)	3.73± (1.47)	<0.001
BMI^a^, Mean ± SD	7,424	29.61± (7.07)	29.63± (7.08)	29.09± (6.73)	0.497
Diabetes^b^, n(%)	7,424				0.450
No		4,659 (67%)	4,546 (67%)	113 (66%)	
Yes		1,320 (13%)	1,278 (13%)	42 (17%)	
Borderline		1,445 (20%)	1,417 (20%)	28 (17%)	
Hypertension^b^, n(%)	7,424				0.001
No		4,630 (67%)	4,551 (68%)	79 (48%)	
Yes		2,794 (33%)	2,690 (32%)	104 (52%)	
Smoking status^b^, n(%)	7,424				0.185
No		6,022 (82%)	5,859 (82%)	163 (87%)	
Yes		1,402 (18%)	1,382 (18%)	20 (13%)	
Drinking status^b^, n(%)	7,424				0.081
No		967 (9.1%)	955 (9.3%)	12 (4.4%)	
Yes		6,457 (91%)	6,286 (91%)	171 (96%)	
WWI^a^, Mean ± SD	7,424	0.60± (0.10)	0.60± (0.10)	0.61± (0.10)	0.154
SIRI^a^, Mean ± SD	7,424	1.27± (0.85)	1.26± (0.83)	1.57± (1.27)	0.016
SII^a^, Mean ± SD	7,424	510.09± (280.06)	508.72± (274.26)	545.84± (402.74)	0.988
Cardiovascular disease^b^, n(%)	7,424				<0.001
No		6,661 (92%)	6,530 (93%)	131 (79%)	
Yes		763 (7.8%)	711 (7.3%)	52 (21%)	
Stroke^b^, n(%)	7,424				0.017
No		7,150 (27%)	6,980 (97.6%)	170 (95%)	
Yes		274 (2.5%)	261 (2.4%)	13 (5.0%)	
Congestive heart failure^b^, n(%)	7,424				<0.001
No		7,199 (98%)	7,030 (98%)	169 (94%)	
Yes		225 (1.9%)	211 (1.7%)	14 (5.8%)	
Coronary heart disease ^b^, n(%)	7,424				<0.001
No		7,138 (97%)	6,982 (97%)	156 (89%)	
Yes		286 (3.3%)	259 (3.0%)	27 (11%)	
Angina^b^, n(%)	7,424				0.426
No		7,255 (98%)	7,079 (98%)	176 (97%)	
Yes		169 (1.9%)	162 (1.9%)	7 (2.7%)	
Heart attack^b^, n(%)	7,424				<0.001
No		7,113 (97%)	6,952 (97%)	161 (92%)	
Yes		311 (3.0%)	289 (2.8%)	22 (7.8%)	

a: Student t-test, b: Chi-square test, SD: standard deviation.

## Results

### Participant characteristics

Table 1 describes the characteristics of the participants. A total of 7424 participants were included in the study, representing a weighted population of 173,312,738. The mean age was 46.84 years and included 3646 males (49%) and 3778 females ((51%)). Of these participants, 183 (2.5%) were diagnosed with NMSC. Patients with NMSC were typically older (age > 49, 89% vs. 43%, p < 0.001), predominantly of other races (p < 0.001), more educated (p = 0.012), and had higher household income (3.73± (1.47) vs. 3.04± (1.64), p < 0.001), higher blood pressure (52% vs. 32%, p = 0.001), higher likelihood of comorbid cardiovascular disease (21% vs. 7.3%, p < 0.001) and its subgroups (except for Angina, p > 0.05), including stroke (5% vs. 2.4%, p = 0.017), congestive heart failure (5.8% vs. 1.7%, p < 0.001), coronary heart disease (11% vs. 3%, p < 0.001), heart attack (7.8% vs. 2.9%, p < 0.001). However, no significant differences were observed in gender, BMI, WWI, SII, diabetes, smoking status and drinking status (p > 0.05).

### Relationship between CVD and its subgroups and NMSC

**Fig 2:**
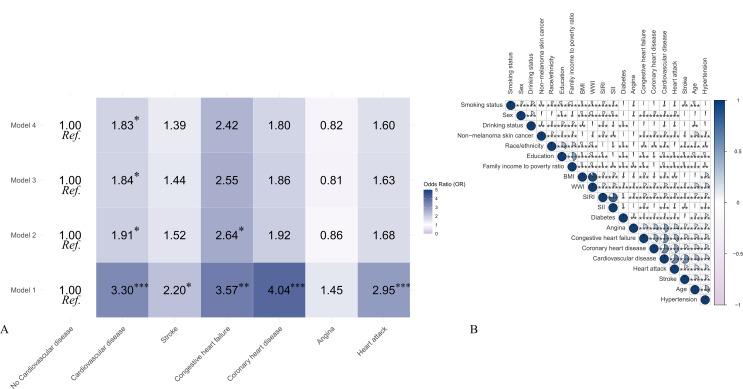
Heatmap of the correlation between CVD and its subgroups and NMSC. Model 1: non-adjusted; Model 2: adjust for sex, age, race/ethnicity, education, family income to poverty ratio, BMI; Model 3: Adjust for sex, age, race/ethnicity, education, family income to poverty ratio, BMI, drinking status, smoking status, hypertension, diabetes; Model4: Adjust for sex, age, race/ethnicity, education, family income to poverty ratio, BMI, drinking status, smoking status, hypertension, diabetes, SIRI, SII, WWI. *P value <0.05, **P value<0.01, ***P value< 0.001.

The association between CVD and its subgroups and the prevalence of NMSC is depicted in Fig 2 and Table 2. Patients with CVD have a 230% higher prevalence of NMSC than patients without CVD (OR=3.3, 95% CI = 2.16 ~ 5.13). The prevalence of NMSC is increased by 230% in patients with CVD compared to those without CVD (OR=3.3, 95% CI = 2.16 ~ 5.13). In the fully adjusted model, adjust for sex, age, race/ethnicity, education, family income to poverty ratio, BMI, drinking status, smoking status, hypertension, diabetes, SIRI, SII, WWI, the above correlation remained significant, with an 83% increase in the prevalence of NMSC in patients with combined CVD (OR=1.83, 95% CI = 1.01 ~ 3.34). Subsequently, we grouped CVD, and neither the original model nor the fully adjusted model showed a significant correlation between the prevalence of NMSC and the CVD subgroups, which included angina, stroke, congestive heart failure, coronary heart disease, and heart attack (P ＞ 0.05).

**Table 2:** Correlation between CVD and its subgroups and NMSC of the NHANES 2015–2018 participants.

Variables	Model1		Model2		Model3		Model4	
	OR (95%CI)	*P*	OR (95%CI)	*P*	OR (95%CI)	*P*	OR (95%CI)	*P*
Cardiovascular disease								
No	1.00 (Reference)		1.00 (Reference)		1.00 (Reference)		1.00 (Reference)	
Yes	3.30 (2.16 ~ 5.03)	<.001	1.91 (1.12 ~ 3.24)	0.019	1.84 (1.01 ~ 3.32)	0.045	1.83 (1.01 ~ 3.34)	0.048
Stroke								
No	1.00 (Reference)		1.00 (Reference)		1.00 (Reference)		1.00 (Reference)	
Yes	2.20 (1.15 ~ 4.22)	0.019	1.52 (0.75 ~ 3.08)	0.227	1.44 (0.69 ~ 3.01)	0.312	1.39 (0.65 ~ 2.98)	0.363
Congestive heart failure								
No	1.00 (Reference)		1.00 (Reference)		1.00 (Reference)		1.00 (Reference)	
Yes	3.57 (1.69 ~ 7.53)	0.001	2.64 (1.06 ~ 6.55)	0.037	2.55 (0.97 ~ 6.72)	0.056	2.42 (0.85 ~ 6.79)	0.087
Coronary heart disease								
No	1.00 (Reference)		1.00 (Reference)		1.00 (Reference)		1.00 (Reference)	
Yes	4.04 (2.14 ~ 7.60)	<.001	1.92 (0.96 ~ 3.85)	0.062	1.86 (0.90 ~ 3.81)	0.086	1.80 (0.88 ~ 3.70)	0.098
Angina								
No	1.00 (Reference)		1.00 (Reference)		1.00 (Reference)		1.00 (Reference)	
Yes	1.45 (0.56 ~ 3.69)	0.428	0.86 (0.28 ~ 2.64)	0.794	0.81 (0.23 ~ 2.88)	0.735	0.82 (0.21 ~ 3.11)	0.751
Heart attack								
No	1.00 (Reference)		1.00 (Reference)		1.00 (Reference)		1.00 (Reference)	
Yes	2.95 (1.63 ~ 5.32)	<.001	1.68 (0.82 ~ 3.47)	0.147	1.63 (0.72 ~ 3.70)	0.222	1.65 (0.72 ~ 3.78)	0.208

OR: Odds Ratio, CI: Confidence Interval.

Model1: Crude.

Model2: Adjust: Sex, Age, Race/ethnicity, Education, Family income to poverty ratio, BMI.

Model3: Adjust: Sex, Age, Race/ethnicity, Education, Family income to poverty ratio, BMI, Drinking status, Smoking status, Hypertension, Diabetes.

Model4: Adjust: Sex, Age, Race/ethnicity, Education, Family income to poverty ratio, BMI, Drinking status, Smoking status, Hypertension, Diabetes, SIRI, SII, WWI.

### Subgroup analysis

**Fig 3:**
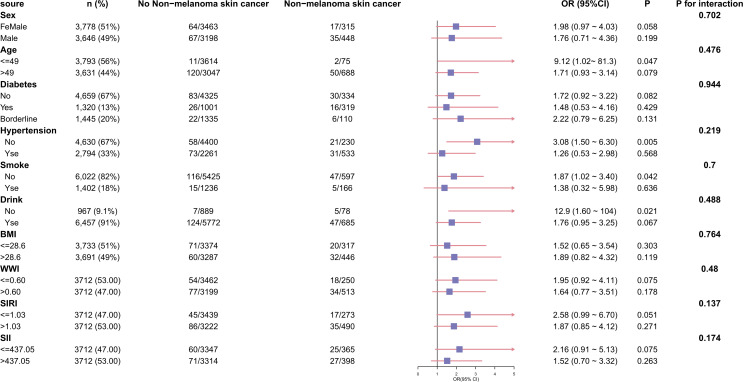
Forest plot depicting subgroup analysis of the association between CVD and NMSC. Race/ethnicity, education and family income to poverty ratio were adjusted. P for interaction >0.05 for all subgroups.

To further demonstrate the relationship between CVD and the prevalence of NMSC, we performed a subgroup analysis of the participants, classifying them by gender, age, diabetes, hypertension, smoking status, drinking status, BMI, WWI, SII, and SIRI (Fig 3 and Table 3). As indicated in Fig 3 and Table 3, CVD and NMSC were still positively correlated among people under 49, without hypertension, non-smoking, and non-drinking (p both < 0.05). However, there was not any significant interaction between CVD and the above-mentioned variables (p for interaction > 0.05), suggesting that the association between CVD and NMSC was consistent across the overall population and across population settings.

**Table 3:** Subgroup analysis of the relationship between CVD and NMSC.

Variables	n (%)	0	1	OR (95%CI)	*P*	P for interaction
Sex						0.702
FeMale	3,778 (51%)	64/3463	17/315	1.98 (0.97 ~ 4.03)	0.058	
Male	3,646 (49%)	67/3198	35/448	1.76 (0.71 ~ 4.36)	0.199	
Age						0.476
<=49	3,793 (56%)	11/3614	2/75	9.12 (1.02 ~ 81.3)	0.047	
> 49	3,631 (44%)	120/3047	50/688	1.71 (0.93 ~ 3.14)	0.079	
Diabetes						0.944
No	4,659 (67%)	83/4325	30/334	1.72 (0.92 ~ 3.22)	0.082	
Yes	1,320 (13%)	26/1001	16/319	1.48 (0.53 ~ 4.16)	0.429	
Borderline	1,445 (20%)	22/1335	6/110	2.22 (0.79 ~ 6.25)	0.131	
Hypertension						0.219
No	4,630 (67%)	58/4400	21/230	3.08 (1.50 ~ 6.30)	0.005	
Yes	2,794 (33%)	73/2261	31/533	1.26 (0.53 ~ 2.98)	0.568	
Smoke						0.700
No	6,022 (82%)	116/5425	47/597	1.87 (1.02 ~ 3.40)	0.042	
Yes	1,402 (18%)	15/1236	5/166	1.38 (0.32 ~ 5.98)	0.636	
Drink						0.488
No	967 (9.1%)	7/889	5/78	12.9 (1.60 ~ 104)	0.021	
Yes	6,457 (91%)	124/5772	47/685	1.76 (0.95 ~ 3.25)	0.067	
BMI						0.764
<=28.6	3,733 (51%)	71/3374	20/317	1.52 (0.65 ~ 3.54)	0.303	
> 28.6	3,691 (49%)	60/3287	32/446	1.89 (0.82 ~ 4.32)	0.119	
WWI						0.480
<=0.60	3712 (53.00)	54/3462	18/250	1.95 (0.92 ~ 4.11)	0.075	
> 0.60	3712 (47.00)	77/3199	34/513	1.64 (0.77 ~ 3.51)	0.178	
SIRI						0.137
<=1.03	3712 (47.00)	45/3439	17/273	2.58 (0.99 ~ 6.70)	0.051	
> 1.03	3712 (53.00)	86/3222	35/490	1.87 (0.85 ~ 4.12)	0.271	
SII						0.174
<=437.05	3712 (47.00)	60/3347	25/365	2.16 (0.91 ~ 5.13)	0.075	
> 437.05	3712 (53.00)	71/3314	27/398	1.52 (0.70 ~ 3.32)	0.263	

OR: Odds Ratio, CI: Confidence Interval.

### Pearson correlation analysis

In order to assess the correlation between CVD, NMSC, BMI, WWI, SII, and SIRI two by two, we used Pearson correlation analysis to further investigate. As shown in Table 4, CVD was positively correlated with NMSC (r = 0.095, p < 0.01), BMI (r = 0.069, p < 0.01), WWI (r = 0.412, p < 0.01), and SIRI (r = 0.016, p < 0.01). There was a positive correlation between NCMS and WWI (r = 0.032, p < 0.01), SIRI (r = 0.067, p < 0.01), and a negative correlation with BMI (r = −0.007, p > 0.05). BMI and WWI (r = 0.911, p < 0.01), SIRI (r = 0.125, p < 0.01), and SII (r = 0.144, p < 0.01) were positively correlated. There was a positive association between WWI and SIRI (r = 0.174, p < 0.01), SII (r = 0.152, p < 0.01), and BMI (r = 0.911, p < 0.01), as well as between SIRI (r = 0.125, p < 0.01) and SII (r = 0.144, p < 0.01). SIRI and SII had a favorable connection (r = 0.771, p < 0.01).

**Table 4:** Bivariate correlation matrix for CVD, NMSC, SII, SIRI, WWI and BMI.

Variables	Cardiovascular disease	Non-melanoma skin cancer	BMI	WWI	SIRI	SII
Cardiovascular disease	1					
Non-melanoma skin cancer	0.095^**^	1				
BMI	0.069^**^	−0.007	1			
WWI	0.142^**^	0.032^**^	0.911^**^	1		
SIRI	0.116^**^	0.067^**^	0.125^**^	0.174^**^	1	
SII	0.020	0.014	0.114^**^	0.152^**^	0.771^**^	1

Note. p < 0.01^**^.

### Analysis of mediating effects

**Fig 4:**
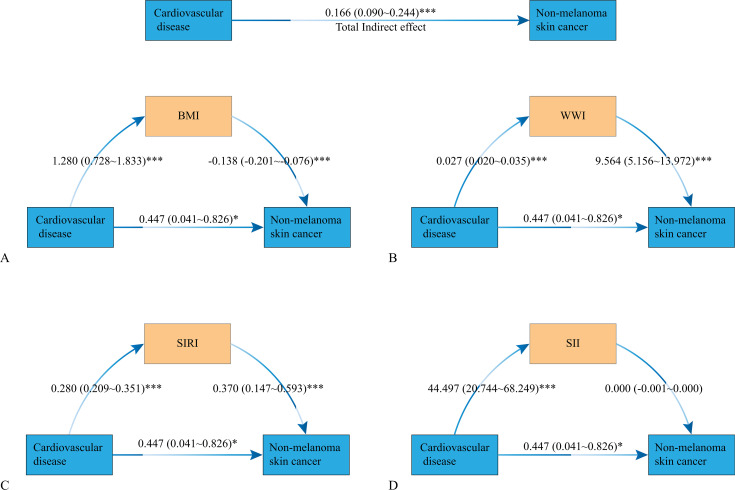
Mediation analysis of BMI, WWI, SII and SIRI in the association between CVD and NMSC. Models were adjusted for sex, age, race/ethnicity, education, family income to poverty ratio, drinking status, smoking status, hypertension, diabetes. *P value <0.05, ***P value< 0.001.

Fig 4 shows the potential mediating role of obesity indices (BMI, WWI) and inflammatory markers (SIRI, SII) in the relationship between CVD and NMSC after adjusting for covariates. We found that higher risk of CVD prevalence was associated with higher BMI (β = 1.280, p < 0.001), WWI (β = 0.027, p < 0.001), SIRI (β = 0.280, p < 0.001), and SII (β = 44.497, p < 0.001). Meanwhile, higher WWI (β = 9.564, p < 0.001), SIRI (β = 0.370, p < 0.001), and lower BMI (β = −0.138, p < 0.001) were linked to a higher likelihood of NMSC prevalence, whereas the relationship between SII and NMSC was not statistically significant (p ＞ 0.05).

Then, we used bootstrap methods to evaluate direct, indirect, and total effects in order to better ascertain the effect size and derive 95% confidence intervals (CI). As indicated in Table 5, the prevalence of NMSC was directly impacted by CVD at 0.447 (95% CI: 0.041, 0.826), while BMI, WWI, SIRI, and SII had indirect effects of −0.177 (95% CI: −0.303, −0.082), 0.261 (95% CI: 0.135, 0.415), 0.104 (95% CI: 0.043, 0.171), and −0.021 (95% CI: −0.066, 0.008), respectively. In summary, the association between CVD and NMSC was largely mediated by BMI, WWI, and SIRI, with the exception of SII.

**Table 5:** Bootstrap tests for mediation models.

Paths	Observed Coefficient	Bootstrap test		LLCI	ULCI
		Bootstrap Standard Error	*P*		
Total Indirect effect	0.166	0.039	<.001	0.090	0.244
Indirect effect(via BMI)	−0.177	0.05	<.001	−0.303	−0.082
Indirect effect(via WWI)	0.261	0.071	<.001	0.135	0.415
Indirect effect(via SIRI)	0.104	0.032	<.001	0.043	0.171
Indirect effect(via SII)	−0.021	0.019	>0.05	−0.066	0.008
Direct effect	0.447	0.199	<.001	0.041	0.826

LLCI, lower level for confidence interval; ULCI, upper level for confidence interval.

Sex, Age, Race/ethnicity, Education, Family income to poverty ratio, Drinking status, Smoking status, Hypertension, Diabetes were adjusted.

## Discussion

This cross-sectional study is the first to utilize the NHANES database to explore the association between CVD and the risk of NMSC, as well as the potential mediating roles of the metrics BMI, WWI, SII, and SIRI, which represent obesity and inflammation. Logistic regression results indicated that after adjusting for confounders (including mediating variables), patients with comorbid CVD were more likely to develop NMSC than those without CVD, although this was not statistically significant in subgroups with CVD, possibly due to limited subgroup sample sizes reducing statistical power. In addition, mediation analyses showed that BMI, WWI, and SIRI all partially mediated the relationship between CVD and NMSC, whereas SII had no significant mediating effect. The association (p = 0.048) is marginally significant, warranting cautious interpretation and replication; clinically, it suggests integrated CVD-NMSC risk management.

A growing body of research suggests that patients with CVD have a higher risk of developing cancer than the general population [12,37], but this does not include NMSC. This paper is the first to explore the potential relationship between CVD and NMSC, further demonstrating the above. The specific pathogenesis of CVD leading to NMSC is not known. In this paper, Pearson correlation analysis initially suggests that obesity and inflammation may play a role. Mechanistic studies, such as mouse models showing heart failure promotes tumor growth via SerpinA3 [10], suggest causality, but longitudinal human data are needed.

Obese tissues produce adipokines like leptin and IL-6, which promote angiogenesis, deregulate apoptosis, and exacerbate UVB-induced inflammation, contributing to NMSC [38]. A previous study found that patients with coronary artery disease had higher plasma leptin levels, and the number of coronary artery branches involved was positively connected with leptin levels [39]. It is known that leptin is significantly expressed in skin squamous cell carcinoma(SCC) [40], and that tumor size correlates with leptin expression in its stroma [41]. In recent years, there has been a gradual awareness of the influence of the tumor microenvironment in the process of tumorigenesis and development. Leptin can promote angiogenesis by upregulating vascular endothelial growth factor and deregulating apoptosis to promote cancer progression [42]. At the same time, obesity caused by alterations in the leptin pathway is accompanied by abnormal cytokine responses to ultraviolet responsiveness (UVR) [38]. It was shown that UVB-induced inflammation was exacerbated in the skin of obese mice and that the pro-inflammatory cytokines TNF-α, IL-6, and IL-1βwere elevated compared to controls [38], suggesting that obesity-induced inflammation interacts with UVB radiation-induced inflammation to cause skin carcinogenesis. In this paper, we selected BMI and WWI to evaluate obesity status and investigate their mediating role. However, to our surprise, there was a negative correlation between BMI and NMSC. This is consistent with the findings of some studies [43,44]. A possible reason is that obese people spend less time outdoors and accumulate less sun exposure, potentially influenced by skin type variations, and the effect of ultraviolet radiation on NMSC masks the effect of obesity [20]. Meanwhile, BMI as a measure of obesity has limitations because it cannot distinguish between lean body mass and fat mass [22]. WWI is a unique index of obesity that not only responds to centripetal obesity, but is also linearly and positively correlated with cardiometabolic morbidity and mortality [22]. In this paper, there was a positive correlation between WWI and NMSC risk, which is in line with our expectations. More studies are required in the future to further discuss the potential mechanisms of association between obesity and NMSC.

Inflammation may be the common biological pathway linking the two. A dense network of macrophage-dominated tissue-resident leukocytes exists in vascular and myocardial tissues of normal individuals [45], due to signals from vascular endothelial cells to circulating leukocytes via adhesion molecules and chemokines [46]. In contrast, the number and function of tissue-resident cells change in the vessel wall in CVD atherosclerosis [46]. As monocytes, neutrophils, lymphocytes, and M1-type macrophages increase, pro-inflammatory factors (cytokines and chemokines) are released [47–49], damaging DNA through oxidative stress and altering the tissue microenvironment, which permits cells to transform malignantly [50]. In a previous Canakinumab Anti-Inflammatory Thrombosis Outcomes Study (CANTOS), researchers discovered that the use of canakinumab, an interleukin-1β blocker, not only reduced the rate of inflammation and cardiovascular events [51], but also reduced cancer mortality by 40% and the incidence of lung cancer by more than 50% [52]. This obliquely supports the hypothesis that inflammation links CVD to cancer. Considering the above process involving lymphocytes, neutrophils, and monocytes, we used the associated SII and SIRI to assess systemic inflammatory status. The results of mediation analysis showed that SIRI partially mediated the correlation between CVD and NMSC, while SII had no mediating effect, which may indicate that monocytes play a greater role in the above inflammatory process by driving tissue-resident leukocyte networks and chronic inflammation [45–49], but more studies are required to confirm it in the future. The significance of SIRI over SII may be due to the predominant role of monocytes in the immune microenvironment, facilitating chronic inflammation that links CVD to NMSC [45–50]. According to the aforementioned results, the association between CVD and NMSC is mediated by obesity and inflammation, and proper exercise and targeting to control the inflammatory process may be an effective way to treat CVD and NMSC.

This is the first study to examine the association between CVD and NMSC using data from a representative large sample. However, it is undeniable that the study has some limitations. First, this paper is a cross-sectional study that could not determine causality, potentially allowing reverse causation and more prospective studies are needed in the future to establish causality between variables. Second, the diagnoses of CVD and NMSC were determined based on self-reports and questionnaires, which may introduce reporting bias, including potential underestimation of NMSC due to recall errors or asymptomatic cases, may affect prevalence estimates. Finally, despite controlling for important covariates, the effect of unknown confounders could not be excluded.

## Conclusion

The study revealed a positive association between CVD and NMSC prevalence. Mediation analysis further indicated that obesity (WWI, BMI) and inflammation (SIRI) partially mediated this association. The above results suggest the importance of weight control in the prevention of CVD and NMSC. Inflammation can be used as a potential therapeutic target in the future.
